# Plasma insulin concentration increases linearly with body condition in Icelandic horses

**DOI:** 10.1186/s13028-016-0258-8

**Published:** 2016-11-09

**Authors:** Anna Jansson, Gudrun J. Stéfansdóttir, Sveinn Ragnarsson

**Affiliations:** 1 Department of Anatomy, Physiology and Biochemistry, Swedish University of Agricultural Sciences, Box 7011, 750 07 Uppsala, Sweden; 2 Department of Equine Science, Holar University College, 551 Sauðárkrókur, Iceland

**Keywords:** Age, Equine, Concentrate, Feed, Fitness, Gender, Hay, Metabolic, Transport

## Abstract

**Background:**

This study investigated the variation in plasma insulin concentration (PIC) in a group of Icelandic horses in training, considered to be healthy and examined possible relationships between PIC and gender, age, body size, body condition score and management factors such as feed allowance and subjective level of fitness.

**Results:**

Plasma insulin concentration ranged from 0.2 to 13.9 mU/l, body condition score from 2.3 to 4.0 and concentrate allowance from 0 to 4 kg. There was a significant effect of concentrate allowance (*P* = 0.0007) and body condition score (*P* = 0.004) on PIC. For every 1 kg increase in the concentrate allowance, log-PIC increased by 0.26 mU/l. For every 1 unit increase in body condition score, log-PIC increased by 0.45 mU/l. There was no effect of hay allowance, level of fitness, transport time, body size and age on insulin concentration.

**Conclusion:**

Owners of Icelandic horses should be aware that increased body condition elevates PIC, and thereby potentially the risk of laminitis. However, in the group of horses studied, the concentrations were within the range considered normal.

## Findings

The Icelandic horse is recognised as an “easy keeper” breed, with the ability to thrive on limited resources and lower feed intake than other horse breeds of the same size. High plasma insulin concentration (PIC) and laminitis have been associated with the easy keeper type of horse and higher insulin concentrations have been observed in Icelandic horses than in Standardbred horses fed the same diet [1].

Knowledge on the variation in PIC in Icelandic horses and factors that influence it is limited. The aim of the present study was to investigate the variation in PIC in a group of Icelandic horses kept on Iceland and considered healthy. An additional aim was to analyse possible relationships between PIC and gender, age, body size, body condition score and management factors such as daily forage and concentrate allowance and subjective level of fitness.

Data were collected from 201 Icelandic horses (129 mares, 72 stallions, age 4–11 years; Table 1) prior to riding evaluations at an official breed evaluation field test. Body weight of each horse was recorded using an electronic scale (Smartscale 300, Gallagher USA) and horses were allocated a body condition score (BCS) according to a system (scale 1–5) devised for Icelandic horses [2]. Data on withers height were obtained from the breeding database (http://www.worldfengur.com). Before the riding test, a blood sample was taken from the jugular vein by vacuum in chilled lithium heparinised tubes (9 ml, Vacuette®, Greine-bio-one, Austria). Plasma was separated by centrifugation (15 min, 520×*g*, Hettich, Tuttlingen, Germany) and stored at −18 °C until analysis. PIC was analysed in duplicate by ELISA (Mercodia AB, Uppsala, Sweden) and between-sample variation was <10%.

**Table 1:** Number of stallions and mares of different ages included in the study

Age (years)	Stallions	Mares
4	15	15
5	22	38
6	13	35
7	16	18
8	5	13
9	1	4
10	0	3
11	0	3
Total	72	129

The owner or rider answered a questionnaire about the horse regarding name, age, feeding and travel time to the showground. The rider’s subjective opinion on the level of preparation of the horse for the test (i.e. fitness for the test) was also registered using a visual analogue scale (1–10, where 1 is badly prepared and 10 very well prepared). The study was approved by the National Animal Research Committee of Iceland (approval no. 0611-3501) and data were collected in Hella, in southern Iceland, on 30 May–2 June and 6–9 June 2011.

Statistical analyses were performed using SAS (Statistical Analysis System package 9.4, Inst. Inc., Cary, NC). Normal distribution of the data was tested with residual plots and insulin values were log-transformed before being subjected to ANOVA. Age data were divided into two groups, ≤5 and >5 years. A new body size measure was also calculated by dividing body weight by withers height. Proc MIXED was used and only sex and significant variables were included in the final model:

$${\text{Y}}_{\text{ijkn}} = \,\upmu \, + {\text{a}}_{\text{i}} + {\text{b}}_{\text{j}} +_{{}} {\text{e}}_{\text{k}} + {\text{ a}}_{\text{n}} + {\text{ e}}_{\text{ijkn}},$$

where Y_ijkn_ is the insulin value, µ the mean value, α_i_ the fixed effect of BCS, β_j_ the fixed effect of concentrate allowance, ε_k_ the fixed effects of sex, a_n_ the random effect of day and e_ijkln_ the residuals (e_ijkln_ ~ 0, δ^2^). Proc CORR was used to calculate correlation. Effects were considered significant at *P* < 0.05.

The true PIC ranged from 0.2 to 13.9 mU/l, BCS from 2.3 to 4.0 and daily concentrate allowance from 0 to 4 kg (Table 2). There was a significant effect of concentrate allowance and BCS on PIC (Table 3). For every 1 kg increase in the concentrate allowance log-PIC increased by 0.26 mU/l, while for every 1 unit increase in BCS log-PIC increased by 0.45 mU/l. There was no correlation between BCS and concentrate allowance (r = 0.04, *P* = 0.59). There was no effect of hay allowance, level of fitness, transport time, body size and age on PIC. There was a tendency (*P* < 0.08) for stallions to have higher PIC than mares.

**Table 2:** Mean, minimum and maximum values for plasma insulin concentration (PIC), age, body size, body condition score (BCS), daily feed allowance, transport time and level of preparation in 201 Icelandic horses shown at a breed evaluation field test in Iceland

	Mean ± SD	Minimum	Maximum
PIC (mU/l)	2.3 ± 1.9	0.2	13.9
Age (years)	5.9 ± 1.5	4	11
Body weight (kg)	340 ± 40	290	416
Height (cm)	141 ± 36	135	149
BCS^a^	3.1 ± 0.8	2.3	4
Hay allowance (kg)	5.6 ± 2.2	0	12
Concentrate allowance (kg)	0.7 ± 0.7	0	4
Transport time (min)	30 ± 27	0	180
Preparation^b^	7.3 ± 2.0	2.3	10

^a^Scale from 1 to 5 according to [2]

^b^Scale from 1 to 10, where 1 is not well prepared and 10 very well prepared for the test

**Table 3:** Effects of body condition score, concentrate allowance and sex on log-transformed plasma insulin concentration in 201 Icelandic horses

Effect	Estimate	Standard error	*P*
Body condition score	0.45	0.15	0.004
Concentrate allowance	0.26	0.08	0.0007
Sex	0.20	0.11	0.07

The table shows the solution for fixed effects (body condition, concentrate allowance and sex) using ANOVA and a mixed model

This study showed that in Icelandic horses considered to be fit for a breed evaluation field test, pre-test PIC increased not only due to increased concentrate intake, as could be expected, but also to a considerable extent due to an increase in BCS. The clearly significant results are to some extent surprising, since the conditions were not standardised or controlled, e.g. concentrate allowances were not weighed and horses were fed at different time points before sampling. Feeding a diet including starch-rich concentrate has been shown to significantly elevate PIC for 5 h compared with a forage-only diet [3]. Thus if horses in the present study were fed concentrate a few hours before being transported to the test, the PIC might still be elevated (average transport time was 30 min). The linear correlation between BCS and PIC confirmed findings by Ragnarsson and Jansson [1] of quite a high regression coefficient (R^2^ = 0.65) between BCS and PIC in a group of Icelandic and Standardbred horses [4].

Transport time and level of fitness were included in the present analysis as factors that could potentially affect PIC. Transport could induce muscle activity and exercise is well known to reduce PIC and, depending on the feeding status, can cause a post-exercise elevation [5]. Exercise training can increase insulin sensitivity [6] and thereby reduce PIC. However, these measures seemed not to influence the variation in insulin concentration in the present study. The lack of response might be due to variation in the subjective assessment of the horses’ fitness for the test.

It has been suggested [7] that a cut-off value of 20 mU/l for PIC (i.e. 20 µU/ml analysed by radioimmunoassay Coat-A-Count [Siemens Medical Solutions Diagnostics, Los Angeles, USA]) in horses fasted over-night can be used as an indicator of insulin resistance (and thereby an increased risk of laminitis). None of the horses in the present study were close to this level and the group studied could therefore be considered sound from this perspective. There was a tendency for stallions to have higher PIC than mares, possibly due to a higher concentrate allowance in stallions than in mares (1 vs. 0.6 kg/day, unpublished data). Age had no effect on PIC, contradicting previous findings that the concentration increases with age [8]. However, the significant effect in that study was observed in horses aged 17–20 years, i.e. much older horses than studied here.

In conclusion, it can be assumed that the BCS range observed in the present study (corresponding to approximately 4–7 on a 9-unit scale) is representative for most Icelandic riding horses and therefore the responses in PIC are also representative. Therefore, owners of Icelandic horses should be aware that increased body condition elevates PIC and that this could potentially increase the risk of laminitis. However, no horse in the present study seemed to be at risk.
